# Short term treatment versus long term management of neck and back disability in older adults utilizing spinal manipulative therapy and supervised exercise: a parallel-group randomized clinical trial evaluating relative effectiveness and harms

**DOI:** 10.1186/s12998-014-0026-7

**Published:** 2014-07-23

**Authors:** Corrie Vihstadt, Michele Maiers, Kristine Westrom, Gert Bronfort, Roni Evans, Jan Hartvigsen, Craig Schulz

**Affiliations:** 1 Northwestern Health Sciences University, Wolfe-Harris Center for Clinical Studies, 2501 W 84th Street, Bloomington 55431, MN, USA; 2 University of Minnesota, Center for Spirituality and Healing, Mayo Memorial Building C592, 420 Delaware Street SE, Minneapolis 55455, MN, USA; 3 Institute of Sports Science and Clinical Biomechanics, University of Southern Denmark, Campusvej 55, Odense DK-5230, M, Denmark

**Keywords:** Neck disability, Back disability, Spinal manipulative therapy, Exercise therapy, Older adults, Mixed-methods, Comparative effectiveness

## Abstract

**Background:**

Back and neck disability are frequent in older adults resulting in loss of function and independence. Exercise therapy and manual therapy, like spinal manipulative therapy (SMT), have evidence of short and intermediate term effectiveness for spinal disability in the general population and growing evidence in older adults. For older populations experiencing chronic spinal conditions, long term management may be more appropriate to maintain improvement and minimize the impact of future exacerbations. Research is limited comparing short courses of treatment to long term management of spinal disability.

The primary aim is to compare the relative effectiveness of 12 weeks versus 36 weeks of SMT and supervised rehabilitative exercise (SRE) in older adults with back and neck disability.

**Methods/Design:**

Randomized, mixed-methods, comparative effectiveness trial conducted at a university-affiliated research clinic in the Minneapolis/St. Paul, Minnesota metropolitan area.

**Participants:**

Independently ambulatory community dwelling adults ≥ 65 years of age with back and neck disability of minimum 12 weeks duration (n = 200).

**Interventions:**

12 weeks SMT + SRE or 36 weeks SMT + SRE.

**Randomization:**

Blocked 1:1 allocation; computer generated scheme, concealed in sequentially numbered, opaque, sealed envelopes.

**Blinding:**

Functional outcome examiners are blinded to treatment allocation; physical nature of the treatments prevents blinding of participants and providers to treatment assignment.

**Primary endpoint:**

36 weeks post-randomization.

**Data collection:**

Self-report questionnaires administered at 2 baseline visits and 4, 12, 24, 36, 52, and 78 weeks post-randomization. Primary outcomes include back and neck disability, measured by the Oswestry Disability Index and Neck Disability Index. Secondary outcomes include pain, general health status, improvement, self-efficacy, kinesiophobia, satisfaction, and medication use. Functional outcome assessment occurs at baseline and week 37 for hand grip strength, short physical performance battery, and accelerometry. Individual qualitative interviews are conducted when treatment ends. Data on expectations, falls, side effects, and adverse events are systematically collected.

**Primary analysis:**

Linear mixed-model method for repeated measures to test for between-group differences with baseline values as covariates.

**Discussion:**

Treatments that address the management of spinal disability in older adults may have far reaching implications for patient outcomes, clinical guidelines, and healthcare policy.

**Trial registry:**

www.ClinicalTrials.gov; Identifier: NCT01057706.

## Background

Musculoskeletal complaints such as back and neck pain are common in the general population but are particularly troublesome in older adults [[1]] and into extreme old age [[2]]. In a one to three month time period, approximately 20-35% of older adults report low back pain [[3]–[6]], 5-22% report neck pain [[4]–[6]], and 9-11% suffer concurrent low back and neck pain [[5],[6]]. Chronic musculoskeletal pain and disability are often associated with increased dependence [[1]], decreased physical functioning [[1],[6],[7]], and other co-morbidities [[6],[8]], which can inhibit vital social activities and quality of life [[7]], as well as contribute independently to mortality [[9]]. Healthcare expenditures for back and neck problems have increased with limited improvement in health status [[10],[11]]. A Medicare claims analysis found back pain to be the second most costly chronic non-cancer pain condition; the adjusted cost attributed to back pain per affected member was $2888 annually [[12]]. With nearly 40 million older adults living in the US [[13]], this quickly growing age group [[14]] is projected to double by 2040 [[13]]. Subsequently, investigating conservative non-pharmacological treatments that temper the effects of back and neck problems is an important public health issue [[12]].

In the general population, exercise therapy has demonstrated effectiveness for back and neck pain and disability [[15],[16]], particularly when tailored to the individual. Evidence suggests that older adults who exercise experience reduced risk of disability and functional decline [[17]]. Accordingly, regular exercise is recommended to maintain health and functional ability among older adults [[18]]. Importantly, Hayden et al. found exercise combined with conservative treatment such as manual therapy improved functional outcomes in the general population with chronic low back pain [[15]].

Approximately 11–17% of older Americans seek care from chiropractors annually [[19],[20]]. While a majority of research has focused on short and intermediate term effectiveness of spinal manipulative therapy (SMT) in the general population [[16],[21]], there is a limited, yet growing body of evidence suggesting effectiveness of SMT for back-related disability in older adults [[22],[23]]. Considering that back and neck pain are often chronic in nature and part of a constellation of co-morbidities that impact functional ability [[24]], a long term management approach may be more appropriate to effectively address back and neck disability in older adults. Long term management may aid in maintaining the improvement in functional capacity achieved during a short course of treatment [[25]] and may minimize the impact of future exacerbations [[26]]. This theory is supported by a small study showing that nine months of continued treatment with SMT sustained participants’ improvement in low back pain and disability compared to those receiving only one month of SMT [[27]]; however, the effectiveness of long term management of back and neck disability in older adults has yet to be investigated in a full scale trial [[28]].

### Aims

The primary aim is to compare the effectiveness of 12 versus 36 weeks spinal manipulative therapy and supervised rehabilitative exercise (SMT + SRE) by assessing change in the Neck Disability Index (NDI) and Oswestry Disability Index (ODI) at 36 weeks.

Four secondary aims assess between-group differences in:

1) secondary patient-rated outcome measures at week 36 at 78, including disability at week 78

2) functional outcomes at week 37

3) patients’ perceptions of treatment

4) cost effectiveness and cost utility at weeks 36, 52, and 78. This aim will be described and reported elsewhere.

## Methods/Design

Ethical approval from Northwestern Health Sciences University’s (NWHSU) institutional review board (IRB) was received in October 2009.

### Design & setting

This randomized, observer-blinded, comparative effectiveness trial is being conducted at the Wolfe-Harris Center for Clinical Studies at NWHSU in Bloomington, Minnesota. Notice of privacy practices and written informed consent are secured from all subjects prior to participation.

### Methodological changes to study protocol

The initial study protocol was a three-arm trial, which included a minimal-intervention comparison group of SRE alone for 36 weeks. Slower than projected enrollment and award reductions from the funding agency prompted changes to the study protocol 18 months after recruitment began. A modified study design was proposed by study investigators and approved by the steering committee, funding agency, IRB, and data and safety monitoring board (DSMB). The initial design, modified design, and rationales for each change are described in Table 1. The following three secondary aims were added to use the data collected from those already randomized to the SRE alone group:

**Table 1:** Differences between the initial and modified trial designs and precipitating reasons for the change

**Initial design**	**Modified design**	**Rationale**
Three-arm trial (n = 300)	two-arm trial (n = 200)	Enrollment
• 36 weeks SMT + SRE (n = 88)	• 36 weeks SMT + SRE (n = 100)	Funding
• 12 weeks SMT + SRE (n = 124)	• 12 weeks SMT + SRE (n = 100)	
• 36 weeks SRE alone (n = 88)		
n = 300	n = 200 (for primary and secondary aims 1–4)	Enrollment
	n = 18 (for additional secondary aims A-C)	Funding
Randomization ratio 1:1.4:1	Final randomization ratio 1:1	Enrollment
		Funding
Pertinent inclusion/exclusion criteria:	Pertinent inclusion/exclusion criteria:	Enrollment
• NDI and ODI ≥ 15% each at both baseline evaluations	• NDI and ODI ≥ 10% each *AND* combined score of ≥ 25% at first baseline evaluation only	
Functional outcomes:	Functional outcomes:	Funding
• short physical performance battery		
• hand grip strength		
• accelerometry	• short physical performance battery	
• postural sway	• hand grip strength	
• range of motion	• accelerometry	
• static endurance		

A) assess within group change for all patient-rated outcomes at weeks 36 and 78

B) assess within group change for functional outcomes at week 37

C) describe participants’ perceptions of treatment

The remainder of this manuscript describes the methodology for the modified trial.

### Participants

**Figure 1:**
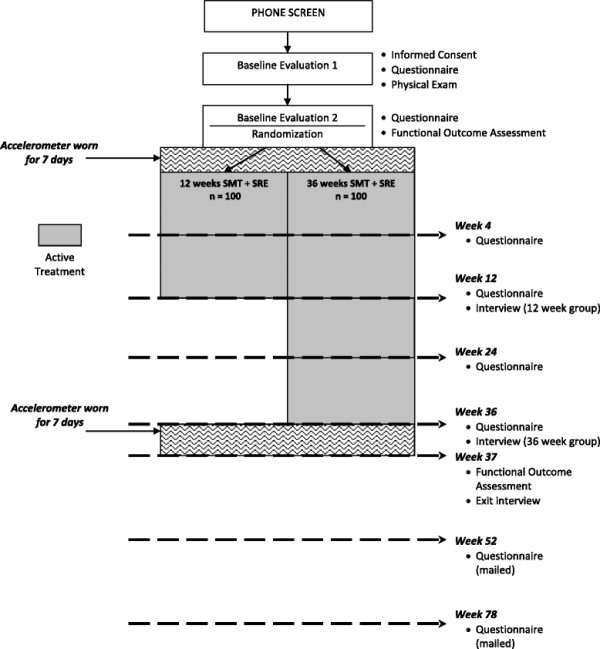
Participant flow.

This study will enroll 200 older adults who report functional disability in the back and neck regions. See Figure 1 for participant flow through the study.

### Recruitment strategy

Participants from the Minneapolis/St. Paul metropolitan area are recruited through targeted mailing of brochures, church bulletins, movie theater advertisements, distributed flyers and posters, informational presentations, letters to physicians, and online strategies such as the NWHSU’s website, Craigslist®, and Facebook®.

### Inclusion/exclusion criteria

To be eligible, participants need to be 65 years of age or older with self-reported back and neck disability.

Inclusion criteria are:

• Back and neck disability of ≥ 12 weeks duration

• Back and neck disability, defined as

◦ 10% or higher on Neck Disability Index (NDI),

◦ 10% or higher on Oswestry Disability Index (ODI), and

◦ A combined score (NDI + ODI) of at least 25%

• Stable pain medication plan 4 weeks prior to baseline

• Ability to read and speak English

• Community dwelling

• Ability to ambulate without the aid of a wheelchair or motorized scooter

Exclusion criteria are:

• Surgical spinal fusion [[29]]

• Multiple incidents of spinal surgery [[29]]

• Spinal surgery in the last 6 months

• Ongoing non-pharmacological treatment for a spinal condition

• Less than 25 on the Folstein Mini-Mental State Examination [[30],[31]]

• Untreated or unstable clinical depression screened by the Geriatric Depression Scale [[32]–[34]]

• Current or pending financial compensation for a neck or back condition [[35],[36]]

• Co-morbid conditions

◦ Ongoing substance abuse

◦ Body mass index ≥ 40

◦ Stage III or IV cancer diagnosis in the past 5 years or active cancer treatment

◦ Uncontrolled hypertension

◦ Advanced Parkinson’s Disease

◦ Advanced multiple sclerosis

◦ Uncontrolled metabolic disease

◦ Diffuse idiopathic skeletal hyperostosis

• Contraindication to SMT

◦ Advanced spinal stenosis [[37]]

◦ Spinal fracture [[37]]

◦ Stroke or transient ischemic attack

◦ Inflammatory or destructive tissue changes of spine

◦ Bleeding disorder [[38]]

◦ Severe osteoporosis [[38]]

◦ Progressive neurological deficits or cauda equina syndrome [[38]]

• Contraindication to SRE

◦ Advanced cardiovascular or pulmonary disease [[39]]

### Eligibility determination

#### Phone screen

Certified study personnel administer a computer-guided questionnaire to interested individuals by phone. Responses are directly entered into a computer program that determines general eligibility for the first baseline evaluation. Baseline evaluation consists of two visits, 7–21 days apart, which serve as a compliance check and provide a more accurate baseline estimate.

#### First baseline evaluation

The first baseline evaluation includes informed consent, cognitive function assessment, self-report questionnaire including demographic and outcome measures, and a history and examination by a licensed clinician. The physical examination focuses on the cervical and lumbar spine and assesses posture, gait, range of motion, orthopedic and neurologic tests. Unless recent imaging is available, cervical and lumbar plain radiographs and bone mineral density scans of the distal radius and ulna are taken to rule out exclusionary co-morbidities and contraindications (e.g., spinal stenosis, osteoporosis). Potential participants that qualify at the first baseline evaluation are reviewed by a group of clinicians and investigators who reach consensus on eligibility (qualify, does not qualify, or referral if necessary).

#### Second baseline evaluation

A second baseline evaluation includes an informed consent (i.e., review of study activities), a self-report questionnaire, and functional outcome assessment. Consenting participants are then randomly allocated by staff masked to upcoming treatment assignment.

### Randomization

Restricted randomization employs a 1:1 allocation ratio. The randomization scheme was generated by an independent statistician using randomly permuted block sizes created with a computerized random number generator. As participants become eligible for randomization, sequentially numbered, opaque, sealed envelopes containing treatment assignments are drawn and opened by study staff in the participant’s presence. The randomization scheme and block sizes are concealed from all study staff including those who take part in eligibility determination, enrollment, and randomization.

### Blinding

Functional outcome examiners are blinded to participant treatment assignment. The physical nature of the treatments prevents blinding of participants and providers to treatment assignment.

### Interventions

Participants receive both SMT and SRE for either 12 weeks or 36 weeks. All are requested to abstain from seeking treatment for their back or neck outside the study. Treatment protocols are based on previous studies by the investigators [[40]] and study clinicians input. The approach to treatment is pragmatic in nature intended to reflect real-world, patient-centered practice and is tailored to participants’ age, physical condition, and preferences [[23]]. Standardized forms are used to document treatment procedures, adverse events, and participant compliance; all forms are reviewed daily for completeness and protocol compliance. See Table 2 for descriptions of the interventions.

**Table 2:** Descriptions of the interventions

**Intervention**	**Type**	**Program design**	**Delivery method**	**Dose**
SMT	High velocity, low amplitude manipulation (can be drop-table assisted) [[41]]	Individualized: spinal regions treated and type of therapy used is determined by the chiropractor [[42],[43]]	Treatment delivered by licensed chiropractors with at least 5 years experience	20 to 30 minute visits
				Minimum: 1 visit/month
	Low velocity, low amplitude mobilization			Maximum: 2 visits/week
				Individualized: Number of treatments determined by chiropractor & patient [[40],[44],[45]]
	Manual distraction, gentle soft tissue massage, hot or cold therapy, and active or passive muscle stretching to facilitate SMT			
SRE	Aerobic warm up	Partially individualized: exercise selection, progression, and repetitions are determined by patient’s abilities and tolerance	Supervised by exercise therapists*	45 to 60 minute sessions
	Stretching, strengthening, and balance exercises			
				Minimum: 1 session/month
			One-on-one	
				Home exercise encouraged between sessions
	Advice to stay active; self-care tips for pain management [[46],[47]]		Uses resistance bands, stability trainers, chairs	

*Exercise therapists are under the supervision of treating chiropractors.

### Spinal manipulative therapy (SMT)

SMT and mobilization are defined as the application of manual force to the spinal joints. Each SMT appointment is approximately 20 to 30 minutes and includes history, examination, and treatment. Appointments focus on complaints of the cervical and lumbar spine; however, other musculoskeletal complaints may be addressed if it impacts spine-related disability (e.g., hip complaint can be addressed if it impacts low back). Treatment areas are identified by palpatory spine tenderness [[42]], decreased vertebral motion, abnormal joint play, or abnormal end feel determined by passive motion tests [[43]]. Treatment procedures include high velocity low amplitude thrust, which can be drop-table assisted [[41]], and low velocity low amplitude mobilization. Manual distraction, gentle soft tissue massage, hot or cold therapy, and active or passive muscle stretching can be used to facilitate or as an adjunct to SMT.

### Frequency of SMT treatment

The minimum frequency of SMT appointments is one per month with a maximum of two per week. The number and frequency of appointments are determined by the chiropractor and patient, guided by responses to a modified version of the Measure Yourself Medical Outcome Profile [[44],[45]].

### Supervised rehabilitative exercise (SRE)

Exercise therapy is defined as progressive stretching, strengthening, and balance exercises, which use resistance bands and stability trainers. SRE sessions are delivered by exercise therapists, under the supervision of chiropractors, in individual 45 to 60 minute sessions. There are 4 sessions spaced throughout the 12 week intervention and 10 sessions in the 36 week intervention. Exercises focus on increasing spinal mobility, strengthening supporting spinal musculature, and increasing overall stability and proprioception (see Table 3). All sessions include a 5 to 10 minute aerobic warm up on a treadmill or stationary bike. At the first session, participants are given information about their spine-related condition, self-care tips for pain management, and benefits of exercise for back and neck problems. Participants review goals of the SRE program and set personal activity goals with their therapist. During the second session, strengthening exercises and body mechanics for activities of daily living are introduced. Exercises are introduced at an intensity commensurate to the participant’s level of fitness and abilities based upon the therapist’s assessment. Subsequent sessions review previous exercises and check for proper form. Ongoing encouragement to promote physical activity and movement to decrease fear avoidance is provided [[46],[47]].

**Table 3:** Details of exercises in SRE program

**Type (Freq)**	**Exercise**	**Sets**	**Repetitions**	**Exercise description**	**Progressions**
Stretching (performed daily at home)					
	head retraction	1	5	Seated with head in neutral position, alternate retracting head back and returning to neutral.	
	cat camel	1	5	Begin with the pelvis in a neutral position, alternate between arching the back in a “C” shape forward and backward.	1: Seated
					2: Hands/knees
	shoulder shrug	1	1	Seated with head and neck in neutral position, raise shoulders in a cephalic direction, and release.	
	neck forward bend	1	1	Seated with head in neutral position, flex head forward to bring chin toward the chest.	
	neck side bend	1	1	Seated with head in neutral position, keep shoulders stationary, tip head to side approximating ear to shoulder. Release & repeat on other side.	
	hamstring stretch	1	1	Seated with one leg straight and one leg bent, flex at the waist while keeping leg straight. Repeat on other side.	
	seated hip stretch	1	1	Seated, place one ankle on the opposite knee. Use the hand to add pressure on bent knee to externally rotate the hip. Repeat on other side.	
Balance (performed daily at home)					
	knee lift	up to 2	up to 5	Stand next to a chair; bend one knee to lift the foot a few inches off the floor; slowly lower foot to floor. Repeat on other side.	1: Chair assisted
					2: Stability trainer, chair assisted
					3: Unassisted
					4: Stability trainer, unassisted
	stance/lunge	up to 2	up to 5	Stance: Stand with feet together, step forward so heel touches the opposite foot’s toes. Return to the start position; repeat on other side.	1: Semi-tandem stance, chair assisted
					2: Semi-tandem stance, unassisted
					3: Stability trainer, semi-tandem stance, chair assisted
					4: Stability trainer, semi-tandem stance, unassisted
					5: Semi-tandem lunge, chair assisted
					6: Stability trainer, semi-tandem lunge, chair assisted
					7: Tandem stance, chair assisted*
					8: Tandem stance, unassisted*
					9: Stability trainer, tandem stance, chair assisted*
					10: Stability trainer, tandem stance, unassisted*
				Lunge: Begin with feet together, take an exaggerated step forward. Lower the knee of the back leg towards the ground and then rise to return to starting position with the feet together. Repeat on other side.	
Strengthening (performed every other day at home)					
	bird dog	up to 2	up to 5	Begin on hands and knees, extend either one or two (contra lateral) extremities parallel to floor. Return to start position; repeat on other side.	1: Hands/knees leg only
					2: Hands/knees, leg and arm combined
					3: Stability trainer, hands/knees, leg and arm combined
	push up	up to 2	up to 10	From a plank position, lower the body by bending the arms, keeping the back straight. Return to start position.	1: Wall
					2: Kneeling
					3: Full body
	abdominal curl	up to 2	up to 10	Lie face up on floor with one knee bent and one leg straight; lift the shoulders off the ground and flex at the waist; release. After first set, switch bent knee.	1: Supine on floor, hands at side
					2: Supine on floor, arms on chest
					3: Supine on floor, hands behind head
					4: Supine on floor, hands overhead
					5: Stability
					trainer, hands at side
					6: Stability trainer, arms on chest
					7: Stability trainer, hands behind head
					8: Stability trainer hands overhead
	resisted head retraction	up to 2	up to 10	Seated with head in neutral position facing a closed door. A resistance band is looped around the head/forehead with end secured by a firmly closed door. Alternate head retraction and release.	1: Yellow Theraband®
					2: Red Theraband®
					3: Green Theraband®
	chair squat	up to 2	up to 10	Stand in front of a chair, bend knees and hips to lower body to a seated position; return to standing.	1: Two handed assist
					2: Arms at sides
					3: Arms crossed
					4: Arms out front
					5: Stability trainer, arms at sides
					6: Stability trainer, arms crossed
					7: Stability trainer, arms out front

*Only one set is required to progress.

Sets and repetitions listed are for each side, if appropriate. Progressions are introduced when the participant can complete the maximum number of sets and repetitions with proper form. Neutral position of the head implies a relaxed posture, the ears aligned with the shoulders. Neutral pelvis cues the patient to position themselves with a slight, not exaggerated, lordosis in the lumbar spine.

Participants are encouraged to perform the exercises at home between supervised sessions (see Table 3). To encourage compliance with home exercise, exercise logs, resistance bands, stability trainers, and exercise handouts are provided. The handouts feature pictures of older adults performing the exercise with simple written instructions.

### Compliance

To be considered compliant in the 36 week group, participants must attend one SMT appointment per month for eight of the nine months and eight of ten SRE sessions. Compliance in the 12 week group is defined as attending one SMT appointment per month for all three months and three of four SRE sessions.

### Rescue medication & reasons for withdrawal

For individuals experiencing acute exacerbation of pain, rescue medications are available by prescription from a study medical doctor following an evidence-based protocol. If a participant becomes involved with litigation for a neck- or back-related condition, demonstrates progressive neurological signs, or develops any co-morbidity that increases the risk of study participation (e.g., a new transient ischemic attack), they are withdrawn from treatment by the steering committee. Participants with medical conditions that warrant additional follow up and treatment are referred.

### Data collection

Outcome measures are collected through self-report questionnaires, interviews, and blinded functional assessments (see Table 4 for data collection schedule). Patient flow characteristics (i.e., number evaluated, disqualified, etc.) are monitored according to the Consolidated Standards of Reporting Trials (CONSORT) guidelines for standardized reporting of clinical trials [[48]].

**Table 4:** Data collection schedule

	**BEV1**	**BEV2**	**W4**	**W12**	**W24**	**W36**	**W37**	**W52**	**W78**
**Demographics**	**X**								
**Clinical characteristics/physical examination**	**X**								
**Self-report outcome measures**									
Disability: NDI [[49]] and ODI [[50]]	**X**	**X**	**X**	**X**	**X**	**X**		**X**	**X**
Pain: 11-box scale [[51]]	**X**	**X**	**X**	**X**	**X**	**X**		**X**	**X**
General health: EuroQol EQ-5D [[52]]	**X**	**X**	**X**	**X**	**X**	**X**		**X**	**X**
Improvement [[40],[53],[54]]			**X**	**X**	**X**	**X**		**X**	**X**
Pain Self-Efficacy Questionnaire [[55]]	**X**	**X**	**X**	**X**	**X**	**X**		**X**	**X**
Tampa Scale for Kinesiophobia [[56],[57]]	**X**	**X**	**X**	**X**	**X**	**X**		**X**	**X**
Satisfaction [[40],[53],[54]]			**X**	**X**	**X**	**X**		**X**	**X**
Medication use [[40],[53]]	**X**	**X**	**X**	**X**	**X**	**X**		**X**	**X**
Expectations [[40],[53]]		**X**		**X**	**X**	**X**		**X**	
Falls [[58]]		**X**		**X**	**X**	**X**		**X**	**X**
*Side effects [[40],[59]]			**X**	**X**	**X**	**X**			
Home exercise frequency			**X**	**X**	**X**	**X**		**X**	**X**
Self-reported influence	**X**	**X**	**X**	**X**	**X**	**X**		**X**	**X**
**Functional outcome measures**									
Hand grip strength [[60]]		**X**					**X**		
Short physical performance battery (SPPB) [[61],[62]]		**X**					**X**		
Accelerometry (7 days) [[63]]		**X**				**X**			
**Qualitative data collection**									
Interviews				**X 12wk**		**X 36wk**			

BEV = Baseline evaluation; W = weeks post-randomization; 12wk = 12 week treatment group only; 36wk = 36 week treatment group only.

*Also collected at treatment visits during intervention phase: BL2-W12 for 12wk group; BL2-W36 for 36wk group.

### Primary outcome measures

#### Self-report questionnaires

##### Back and neck disability

The Oswestry Disability Index [[50],[64]] (ODI) version 2.0 (section 4, item 6, has been modified to read “I am in bed most of the time.”) and the Neck Disability Index [[49]] (NDI) are valid and reliable outcome measures for back- and neck-related disability. The NDI was derived from the ODI; therefore, both instruments have similar measurement properties, which may aid in the comparison of results. Each outcome measure has 10 sections, each section with six possible responses that reflect increasing disability (0 = no disability, 5 = maximal disability).

### Secondary outcome measures

#### Self-report questionnaires

##### Pain

Patients with spinal conditions consider pain to be one of the most important outcome measures [[65]]. Participants are asked to rate their typical level of neck, mid back, arm, low back, and leg pain during the past week on an 11-box scale (0 = no pain, 10 = worst pain possible); one response for each area [[51]].

##### General Health

The EuroQol EQ-5D [[52]] is used to determine the participant’s general health state. It is a multi-attribute utility scale that measures five dimensions (mobility, self-care, usual activities, pain/discomfort, anxiety/depression) with three response levels (no problem, moderate problem, severe problem). It also includes a visual analog scale, the EuroQol thermometer, which measures overall health status.

##### Improvement

Improvement in both back and neck problems after starting treatment in the study is measured using a single nine-point ordinal scale (1 = no symptoms/100% improvement, 9 = as bad as it could be/100% worse) [[40],[53],[54]].

##### Self-efficacy

The Pain Self-Efficacy Questionnaire is a valid and reliable [[55]] 10-item scale used to assess the participant’s confidence level (0 = not at all confident, 6 = completely confident) when performing physical and social activities in the presence of chronic pain.

##### Kinesiophobia

The Tampa Scale of Kinesiophobia [[56],[57]] measures fear of movement and (re)injury; it has been shown to be valid and reliable in chronic pain conditions [[66]] including back pain [[67]]. It is a 17-item tool that is scored using a four-point Likert scale (1 = strongly disagree, 4 = strongly agree).

##### Satisfaction

Participants will rate how satisfied they are with the care they have received in the study on a seven-point scale (1 = completely satisfied/couldn’t be better, 7 = completely dissatisfied, couldn’t be worse) [[40],[53],[54]].

##### Medication Use

Participants report frequency of use for over-the-counter and prescription medications for their back or neck problem during the past week; this is measured using an eight-point scale (0 = have not taken any, 7 = every day). Participants then identify the medications used during the past week [[40],[53]].

Improvement, satisfaction, and medication use outcome measures have not been tested for validity or reliability.

### Functional outcome measures

Functional outcome assessments take approximately 30 minutes and occur at baseline and week 37.

#### Functional ability

##### Hand Grip Strength

Hand grip strength, a surrogate of overall functional ability and mortality [[68]–[70]], measures the grip strength exerted in a maximum effort using a hand-held hydraulic dynamometer (JAMAR Hand Dynamometer, Therapeutic Equipment Corporation, Clifton, NH) [[71],[72]]. The procedure and scoring rubric are from Mathiowetz et al. [[60]]. This test was modified by alternating hands between each measurement; further, it is considered invalid if the participant cannot perform 3 or more contractions or if there is more than a 20 kg difference between any two measurements.

##### Short Physical Performance Battery (SPPB)

The SPPB is shown to predict future disability in healthy community dwelling older adults over the age of 70 [[62]]. Adapted from the National Institute on Aging, it is comprised of three tests: gait speed, standing balance, and chair rising [[61]]. Each component of the SPPB is scored on a five-point scale (0 = inability to perform, 4 = highest level of performance); these are summed to produce a composite score. The protocols and scoring rubrics are based on the method developed by Guralnik et al. [[62]].

Modifications of the SPPB include reordering of the tests and performing tests on a force plate. Specifically, gait speed is performed first with the shoes on, while the two remaining components are completed in stocking feet on the force plate. The force plate records ground reaction forces during the standing balance and chair stand tests (Bertec Force Plate, Model #4060-NC, Bertec, Inc, Columbus, OH) using Motion Monitor data acquisition software (Innovative Sports Training, Inc, Chicago, IL) to define the participant’s center-of-pressure.

#### Physical activity

##### Accelerometry

A valid and reliable measure for physical activity is an accelerometer [[63]], which measures activity in three planes including the intensity and duration of movement. The GT3X accelerometer (Actigraph, Inc, Pensacola, FL) is worn for 7 consecutive days at 2 time points: prior to both the first treatment visit and week 37. The GT3X is light (19 grams), small (4.6 x 3.3 x. 2.5 cm), and worn at the hip.

### Qualitative interviews

One-on-one interviews are conducted upon completion of treatment [[73]]. The interview format is semi-structured; trained interviewers follow a standardized protocol for conducting interviews [[74]] beginning with open-ended questions followed by probing questions to elicit underlying reasons and additional details:

 • When you have discomfort or pain in your neck or back, how does it affect you? *(Probes: Can you tell me more about that? In what ways does it affect your life?)*

 • Do you expect your neck and back problems will improve, stay the same, or get worse in the future? *(Probes: In what way?)*

 • In general, when seeking care for your neck and back problems, what types of things make a treatment worthwhile to you? *(Probes: Overall, what do you look for in a treatment? What makes a treatment worth investing your time, energy, or money in?)*

 • In your opinion, what was the most beneficial/helpful part of being in this study? *(Probes: Why is that? Was there anything you liked about the study?)*

 • What was the least beneficial/helpful part of being in this study? *(Probes: Why is that? Was there anything you didn’t like about the study?)*

Interviews are kept confidential to allow the participants to speak freely and audio-recorded if the participant consents [[73]]. Recorded interviews are transcribed for analysis; a portion of the transcriptions are cross-checked with the audio for quality assurance purposes.

### Additional outcome measures

#### Falls

Data on falls is collected through a modified outcome measurement tool [[58]]. Participants are asked if they have fallen and landed on the floor or ground or have fallen and hit an object like a table or chair during the past four weeks. If they respond ‘yes,’ they are asked how many times they have fallen during the past four weeks (1, 2–3, 4–5, or 6 or more) and injuries sustained (broke or fractured bone, hit or injured my head, sprain or strain, bruise or bleeding, some other kind of injury, and no injuries).

#### Side effects

Participants report side effects by indicating ‘yes’ or ‘no’ to the following list of known contraindications and potential side effects of SMT and SRE:

• Increase in neck or back pain

• A different type of pain than usually experienced

• Dizziness or nausea^*^

• Increase in numbness or tingling in the arms/hands^*^

• Increase in numbness or tingling in the legs/feet^*^

• Numbness in the saddle area^*^

• Change in bowel or bladder habits^*^

• Increase in difficulty in lifting one or both feet while walking^*^

• Other

*Triggers a clinical evaluation by a study doctor to further assess the participant’s condition.

For each side effect indicated, the participant rates the bothersomeness of the symptom on an ordinal 11-point scale (0 = not at all bothersome, 10 = extremely bothersome) [[40],[59]].

#### Home exercise

Participants report how frequently they performed the study exercises in the past week; this is measured on an eight-point scale (0 = have not done any, 7 = every day).

These three additional outcome measures have not been tested for validity or reliability.

### Adverse events and unanticipated problems

Expected and unexpected adverse events and unanticipated problems (AE/UP) are captured when possible. Active surveillance of harms [[75]] occurs at every treatment visit through standardized treatment forms (see *Side Effects*). Passive surveillance of harms [[75]] occurs at all time points. AE/UPs are categorized by investigators using a standardized form with categories congruent with U.S. Department of Health and Human Services [[76]]. Reportable AE/UPs are forwarded within 3 business days to the DSMB and funding agency and to the IRB within 10 business days. All AE/UPs are reported unblinded to the DSMB annually and to the IRB upon request.

### Potential confounding variables

Variables that may influence outcomes, such as depression, level of physical activity outside the study setting, expectations to treatment, and other health care utilization, are measured and will be taken into account during the statistical analysis, if appropriate.

#### Depression

The Geriatric Depression Scale (short form) is administered at baseline to screen for depression in older, possibly cognitively impaired populations [[32]–[34]].

#### Physical activity

Participants rate the amount of physical activity outside the study setting in their daily routine at baseline (none, very light, light, moderate, heavy, very heavy).

#### Expectations

Participants are asked just prior to randomization how they expect to respond to both treatment groups (much worse, worse, no change, better, or much better) [[40],[53],[77]]. Participants are also asked how much they expect their back or neck problem to change 3 months from now using a nine-point scale (1 = no symptoms/100% improvement, 9 = as bad as it could be/100% worse) at baseline and at weeks 12, 24, and 36. At week 52, participants are asked how they expect their back and neck problem to be six months from now.

#### Healthcare utilization

This is captured in self-report questionnaires by asking if participants have seen any non-study health providers for their back and neck problem in the last month. Treatment received from non-study providers is also captured in the standardized treatment forms.

### Data analysis

#### Statistical methods

Data analyses will be conducted using SAS for Windows (Release 9.1 or higher). Descriptive statistics will be calculated to describe patient baseline characteristics in each treatment group and to assess comparability and generalizability. Baseline values of self-report outcome variables will be obtained by averaging the two baseline visits. Demographic and clinical variables determined by the investigators to impact outcomes or those that have a correlation of 0.5 or greater will be considered as other possible covariates [[78]]. Intention-to-treat analysis will be used; patients with one or more follow up measures will be included in the analysis. Normality assumptions will be evaluated through normal probability plots and data transformed, if necessary.

### Sample size

Sample size is based on detecting a minimally important between group difference of 10% [[79]] in the ODI at week 36, with a variance of 0.20 [[80],[81]]. Using baseline values as covariates in a two-arm design, 85 subjects per group allows a power of 0.90 to be achieved at an alpha level of 0.025. Assuming a 15% dropout or loss to follow up rate, 100 patients are required per group, for a total of 200 subjects.

### Primary and secondary analyses

Primary analysis will use a linear mixed-model method for repeated data to test for between-group differences in neck and back disability separately at week 36, with baseline variables that may influence outcomes as covariates [[82],[83]]. Secondary analysis of disability will include testing for between-group differences at weeks 4, 12, 24, 52, and 78, as well as within-group change at all time points. Longitudinal analysis will be performed through the short (weeks 4, 12, 24 and 36) and long term (weeks 4, 12, 24, 36, 52 and 78).

Secondary outcome measures, including pain, general health, improvement, self-efficacy, kinesiophobia, satisfaction, medication use, and functional outcome measures will be similarly calculated for within- and between-group differences. This study is not powered to detect change in the secondary outcome measures.

Content analysis of qualitative interviews will use both inductive and deductive approaches [[84]] to identify themes that occur in response to questions asked [[85]]. When coding is complete, frequency of themes will be quantified and representative quotations will be identified [[85],[86]].

### Confirmatory analyses

Additional confirmatory analysis will calculate the area under the curve for each variable, taking into account the increasing time intervals between assessments [[87]]. If the area under the curve analysis differs in result from the repeated measures, it suggests that the cumulative experience over time is different.

## Discussion

This is one of the first full-scale randomized clinical trials to compare short term treatment and long term management using SMT and exercise to treat spine-related disability in older adults. It builds on previous research by the investigative team showing improvement with three months of SMT and exercise in similar populations, which regressed to baseline values in long term follow up without further intervention [[88]]. As back and neck pain in older adults are often chronic and among several co-morbidities [[6],[8]], we theorized that long term management may result in sustained improvement compared to short term treatment. Identifying the most favorable duration of treatment is a pragmatic question common to patients, clinicians, policy makers, and third-party payers alike [[25],[89]]. This is especially important to address in an older population, whose long term functional ability is essential to maintaining vitality and independence.

In addition to effectiveness, this trial systematically evaluates harms associated with SMT and SRE. There is a need to improve the reporting of harms in general [[75]], and in particular, those associated with exercise programs [[90]] and SMT [[26],[91]] where evidence is limited [[92],[93]]. Importantly, for older adults, the harms may be different from those experienced in general population due to the age-related changes and the natural history of other diseases [[90]]. This may cause concern for patients or practitioners, and the current lack of evidence highlights the importance of collecting this data [[23],[89]]. For these reasons, this trial developed and implemented standardized, prospective data collection strategies to systematically report harms associated with SMT and SRE. Improved reporting of harms, in addition to effectiveness, will provide more balanced information on risks and benefits of these treatments, which then can be translated into clinical practice.

The qualitative component of this study explores older adults’ experiences with back and neck problems, a condition which has been widely acknowledged as a complex phenomenon [[94]]. A patient’s individual experience with back and neck problems is difficult to fully appreciate with quantitative data collection alone. Using a mixed-method approach allows this study to better understand the impact of study treatments through complementary approaches to data collection, facilitating a more robust interpretation and understanding of spine-related disability in older adults. Additionally, these results may enlighten the design and implementation of spine care treatment for older adults in both future research studies and clinical practice.

The Patient-Centered Outcomes Research Institute (PCORI) has called for “comparative clinical effectiveness research that will give patients and those who care for them the ability to make better-informed health decisions” [[95]]. Pragmatic study designs reflect real world practice, using input from stakeholders such as clinicians to answer practical questions [[96]]. This trial was designed with that goal in mind. Study clinicians were engaged in developing parameters for the study treatments to help investigators determine protocols for frequency of visits and specific therapies used in the treatment encounter. Further, care was individualized to patients according to their age, physical condition, and preferences. To that end, the interventions in this study are designed to be more reflective of clinical practice and increase the generalizability of results when the study is complete. Subsequently, clinically useful findings from this study may guide health care decisions and policy regarding conservative non-pharmacological management of spinal disability in older adults.

### Trial status and timeline

Recruitment began in January 2010 and was completed in May 2013; participants received treatment through December 2013. Data collection will continue through 2014 which will be followed by data cleaning, analysis, and reporting in 2015.

## Abbreviations

AE/UP: Adverse events and unanticipated problems

CONSORT: Consolidated Standards of Reporting Trials

DSMB: Data and safety monitoring board

HRSA: Health Resources and Services Administration

IRB: Institutional Review Board

NDI: Neck Disability Index

NWHSU: Northwestern Health Sciences University

ODI: Oswestry Disability Index

SMT: Spinal manipulative therapy

SPPB: Short Physical Performance Battery

SRE: Supervised rehabilitative exercise

## Competing interests

The authors declare that they have no competing interests.

## Authors’ contributions

MM, JH, RE, GB, KW, and CS are investigators for this trial; MM, as the principle investigator, has overall responsibility for the conduct of the trial. MM, JH, RE, GB, KW participated in the initial trial concept. MM, JH, RE, GB, KW, and CS contributed to protocol development, implementation and redesign. CV is a research fellow and a project manager for the trial. CV prepared the first draft of the manuscript and organized revisions under the mentorship of MM. All authors read, provided feedback, and approved the final manuscript.
